# Comparative Analysis of Psychophysiological Responses in Fibromyalgia Patients: Evaluating Neuromodulation Alone, Neuromodulation Combined with Virtual Reality, and Exercise Interventions

**DOI:** 10.3390/medicina60030404

**Published:** 2024-02-27

**Authors:** Alejandro Rubio-Zarapuz, María Dolores Apolo-Arenas, Pablo Tomas-Carus, José Francisco Tornero-Aguilera, Vicente Javier Clemente-Suárez, Jose A. Parraca

**Affiliations:** 1 Faculty of Sports Sciences, Universidad Europea de Madrid, Tajo Street, s/n, 28670 Madrid, Spain; alejandro.rubio@universidadeuropea.es (A.R.-Z.); vicentejavier.clemente@universidadeuropea.es (V.J.C.-S.); 2 Department of Medical Surgical-Therapy, Faculty of Medicine and Health Sciences, Universidad de Extremadura, 06006 Badajoz, Spain; mdapolo@unex.es; 3 Research Group PhysioH, University of Extremadura, 06006 Badajoz, Spain; 4 Departamento de Desporto e Saúde, Escola de Saúde e Desenvolvimento Humano, Universidade de Évora, 7004-516 Évora, Portugal; ptc@uevora.pt; 5 Comprehensive Health Research Centre (CHRC), University of Évora, 7004-516 Évora, Portugal; 6 Grupo de Investigación en Cultura, Educación y Sociedad, Universidad de la Costa, Barranquilla 080002, Colombia

**Keywords:** fibromyalgia innovations, pain modulation, virtual rehabilitation, exercise therapy, electrostimulation, pain management, psychophysiological assessment

## Abstract

Background and Objectives: Fibromyalgia, a chronic condition, manifests as widespread musculoskeletal pain, fatigue, sleep disturbances, autonomic and cognitive dysfunction, hypersensitivity to stimuli, and various somatic and psychiatric symptoms. This study, a controlled and randomized experiment, aimed to evaluate and compare the immediate effects of different treatments on fibromyalgia patients. Materials and Methods: The treatments included the EXOPULSE Mollii suit, a combination of the EXOPULSE Mollii suit with a virtual reality (VR) protocol, and a physical exercise regimen. A cohort of 89 female fibromyalgia patients was randomly assigned to one of four groups: Control (*n* = 20), Suit only (*n* = 22), Suit combined with VR (*n* = 21), and Exercise (*n* = 26). Results: This study found notable differences across the groups in several key parameters. In the Control group, significant changes were observed in Forced Expiratory Volume (FEV 1/FEV 6), the Numeric Rating Scale (NRS) for pain, Pressure Pain Threshold (PPT) at the epicondyle, cortical arousal levels, the 10 m up-and-go test, and in all measured variables related to temperature and muscle oxygenation. For the group using the suit alone, there were significant differences noted in the NRS, the chair stand test, palm temperature, and all muscle oxygenation parameters. The Suit + VR group showed significant changes in the NRS, PPT at the knee, handgrip strength test, the 10 m up-and-go test, one-leg balance test with the right leg, muscle oxygen saturation (SmO_2_), deoxygenated hemoglobin (HHb), and oxygenated hemoglobin (O_2_Hb). Finally, the Exercise group exhibited significant differences in FEV 1/FEV 6, chest perimeter difference, NRS, PPT at both the epicondyle and knee, cortical arousal, the chair stand test, the 10-m up-and-go test, and in SmO_2_, HHb, and O_2_Hb levels. Conclusions: combining neuromodulation with VR and targeted exercise regimens can effectively alleviate fibromyalgia symptoms, offering promising avenues for non-pharmacological management.

## 1. Introduction

Fibromyalgia is a chronic condition characterized by widespread musculoskeletal pain accompanied by fatigue, sleep disturbances, autonomic dysfunctions, cognitive impairments, hypersensitivity to external stimuli, and a range of somatic and psychiatric disorders [1,2,3]. Ranking as the third most prevalent musculoskeletal disorder after lumbar pain and osteoarthritis, its global prevalence is estimated at 2–3%, escalating to 4.7% within Western European nations [3,4]. The disorder predominantly affects females over males at a ratio of 3:1 [5] and exhibits an increased prevalence with age, peaking between the ages of 50 and 60 [6]. The nature, location, and intensity of the musculoskeletal pain experienced by fibromyalgia patients can vary greatly, influenced by factors such as occupation, comorbidities, environmental conditions, and both physical and mental stress [7,8,9,10].

Patients frequently report both physical and mental fatigue, ranging from mild tiredness to severe exhaustion akin to that seen in febrile illnesses [11]. Sleep disturbances, particularly non-restorative sleep, are common [12], as are cognitive dysfunctions and memory deficits [13]. Additional symptoms include depression and anxiety [14], clinical manifestations such as headaches, dyspepsia, abdominal pain, and symptoms associated with irritable bowel syndrome and genitourinary disorders [15,16,17,18], along with morning stiffness [19,20]. Autonomic disturbances, including xerostomia, xerophthalmia, blurred vision, and photophobia, are also prevalent [21,22,23]. A generalized state of distress and negative emotions are often observed in fibromyalgia patients, potentially leading to psychiatric disorders, with a reported prevalence of 60% for anxiety disorders and 14–36% for depression, in contrast to 6.6% in the healthy population [24,25,26].

Recent findings also highlight mitochondrial dysfunction in fibromyalgia patients, marked by significantly lower muscle oxygen saturation levels than those observed in the general population, sometimes dropping to as low as 20% compared to the normal level of approximately 75% [1,27,28]. The underlying cause of this reduction, whether it stems from an energy production deficit at the mitochondrial level or an increased energy demand by muscle fibers, remains unclear [27,28]. Given the mitochondria’s critical role in energy production through aerobic metabolism, this dysfunction may contribute significantly to the reduction in functional capabilities observed among fibromyalgia patients. Additionally, time since the diagnosis of fibromyalgia has been shown to significantly influence disease outcomes, affecting symptom severity, treatment response, and overall quality of life. Thus, early diagnosis and intervention could potentially modify the disease’s trajectory, highlighting the importance of timely and accurate identification of fibromyalgia to optimize patient management and improve prognostic outcomes [2,3].

The diagnosis of fibromyalgia presents considerable challenges due to the absence of visible clinical signs, which distinguishes it from other rheumatic conditions, and due to the lack of definitive biomarkers for the disease [29]. Over the past three decades, five distinct sets of classification and diagnostic criteria have emerged for fibromyalgia [3]. Recent advances in the analysis of microRNA, proteome, and metabolome offer promising avenues for disease detection [30]. The etiology of fibromyalgia remains unclear, but it is acknowledged that multiple factors play a role in its onset, including genetic predisposition, significant psychological trauma, peripheral inflammation, and dysregulation of central mechanisms. These elements contribute to dysfunctions in pain processing at both the peripheral and central levels, leading to neuromorphological changes associated with nociplastic pain [3,31,32].

Fibromyalgia’s multifactorial nature necessitates a comprehensive, multidisciplinary treatment approach. The cornerstone of treatment encompasses pharmacological and psychological therapies, patient education, and interventions focusing on exercise and nutrition [33,34]. Pharmacological strategies aim to manage pain, with certain centrally acting medications, such as antidepressants and anticonvulsants, proving effective in modulating pain-inhibitory neurotransmitters, reducing neural horn sensitization, and addressing systemic hyperexcitability [35]. However, only approximately one in four patients achieves a 30% reduction in pain with antidepressant treatment [33,36]. Due to the disease’s variable manifestation among individuals, treatment may also include muscle relaxants [37,38,39,40], analgesics [33,41], hypnotic and antipsychotic drugs [33,42], and cannabinoids [43]. Despite extensive research, no single medication has shown efficacy in more than half of the treated patients [44]. Cognitive–behavioral therapy, emphasizing the development of effective coping strategies, has demonstrated superior benefits in managing pain, physical function, and mood among fibromyalgia patients compared to other therapies [45]. Patient education is vital for helping individuals understand their condition, its chronicity, and the proactive role required in its management [35,46,47,48], encouraging patients to adopt personalized coping strategies to enhance their quality of life [35,46,47,48].

Pharmacological treatments offer a specific approach to the physiological aspects of fibromyalgia, contrasting with the broad-spectrum impact of non-pharmacological treatments. These latter methods, which include spa therapy [49,50,51,52], Tai chi, qigong, and yoga [53,54], mindfulness techniques [55,56,57], hypnosis [58,59], acupuncture [60,61,62], thermal or cryotherapy [52], hyperbaric oxygen therapy [63,64,65], and transcranial electrical and magnetic stimulation [66,67], often provide a multidimensional benefit to patients, a complexity not typically achievable with pharmacology alone [49]. Neuromodulation techniques, such as transcutaneous electrical nerve stimulation (TENS), have proven beneficial in improving pain perception, fatigue, and overall quality of life in fibromyalgia patients [68,69,70,71,72,73,74,75]. Another innovative neuromodulation treatment is the EXOPULSE Mollii^®^ suit (EXONEURAL NETWORK AB, Danderyd, Sweden), a full-body garment with integrated electrodes, which has shown effectiveness in enhancing pain perception, muscle oxygenation, parasympathetic modulation, and functional capabilities in fibromyalgia patients [1,76,77].

In the management of fibromyalgia, a condition characterized by widespread musculoskeletal pain, fatigue, and a myriad of other symptoms, a holistic approach that includes both pharmacological and non-pharmacological interventions is paramount. The initial strategy often recommends prioritizing non-pharmacological interventions, with a particular focus on exercise and dietary modifications, due to their broad-spectrum benefits and minimal side effects [33]. These interventions may include aerobic and strength training exercises, aimed at enhancing physical function and pain management, as well as dietary changes and weight loss strategies to mitigate obesity-induced inflammation and reduce peripheral nociceptive inputs, thereby improving posture and overall well-being [3,33,78]. Aerobic exercise, for instance, has been evidenced to significantly improve pain thresholds and functional abilities in fibromyalgia patients [79].

Thus, the purpose of this study is to investigate the psychophysiological responses of fibromyalgia patients to a 60 min session employing the EXOPULSE Mollii suit, either as a standalone treatment or in combination with a virtual reality (VR) protocol, in comparison to a traditional exercise training session. The EXOPULSE Mollii suit, a novel neuromodulation device, is designed to enhance pain perception, muscle oxygenation, and functional capabilities through integrated electrodes that stimulate various muscle groups. When paired with VR, this intervention may offer an immersive experience that could further modulate pain perception and improve patient outcomes by leveraging the distraction and engagement afforded by virtual environments.

Therefore, the primary hypothesis posits that significant differences in psychophysiological responses, including measures of pain perception, muscle function, and overall quality of life, will be observed between baseline and post-intervention assessments across the different treatment modalities. This hypothesis is grounded in the premise that both the EXOPULSE Mollii suit and VR technology can offer unique benefits to fibromyalgia patients, potentially surpassing those obtained through traditional exercise regimens alone. By integrating advanced technological interventions with conventional treatment strategies, this study aims to elucidate the comparative efficacy of these approaches and contribute to the optimization of fibromyalgia management protocols [1,33,77].

## 2. Materials and Methods

### 2.1. Study Design

The present research unfolded within the Faculty of Medicine at the University of Badajoz, Spain. This randomized, controlled experimental study was meticulously designed to evaluate and compare the immediate effects of three distinct treatment modalities on individuals diagnosed with fibromyalgia, strictly adhering to the 2016 American College of Rheumatology (ACR) Criteria [10].

The interventions under comparison include a treatment session utilizing the EXOPULSE Mollii (Exoneural Network, Sweden) suit in isolation; a hybrid treatment session that combines the EXOPULSE Mollii suit with a virtual reality (VR) protocol; and a control session consisting of conventional training exercises.

Recruitment of participants was actively conducted from September 2022 to December 2022, ensuring a robust and comprehensive enrollment period. The intervention period started in January 2023. This timeframe allowed for a meticulous selection process, ensuring that all participants met the stringent diagnostic criteria for fibromyalgia set forth by the ACR. This study’s structured methodology was designed to rigorously assess the acute responses of fibromyalgia patients to each intervention, with the follow-up period extending to capture the immediate and short-term effects of the treatments.

### 2.2. Participant

Following an extensive period of patient recruitment and evaluation, this study ultimately enrolled 89 female fibromyalgia patients. This number represents the maximum cohort size attainable within the constraints of our available time slots, facility capacities, and staffing resources, in addition to strictly adhering to the predefined inclusion and exclusion criteria detailed in Table 1. This careful selection process ensured the assembled study group fully met the operational and clinical prerequisites for participation.

### 2.3. Intervention

**Figure 1:**
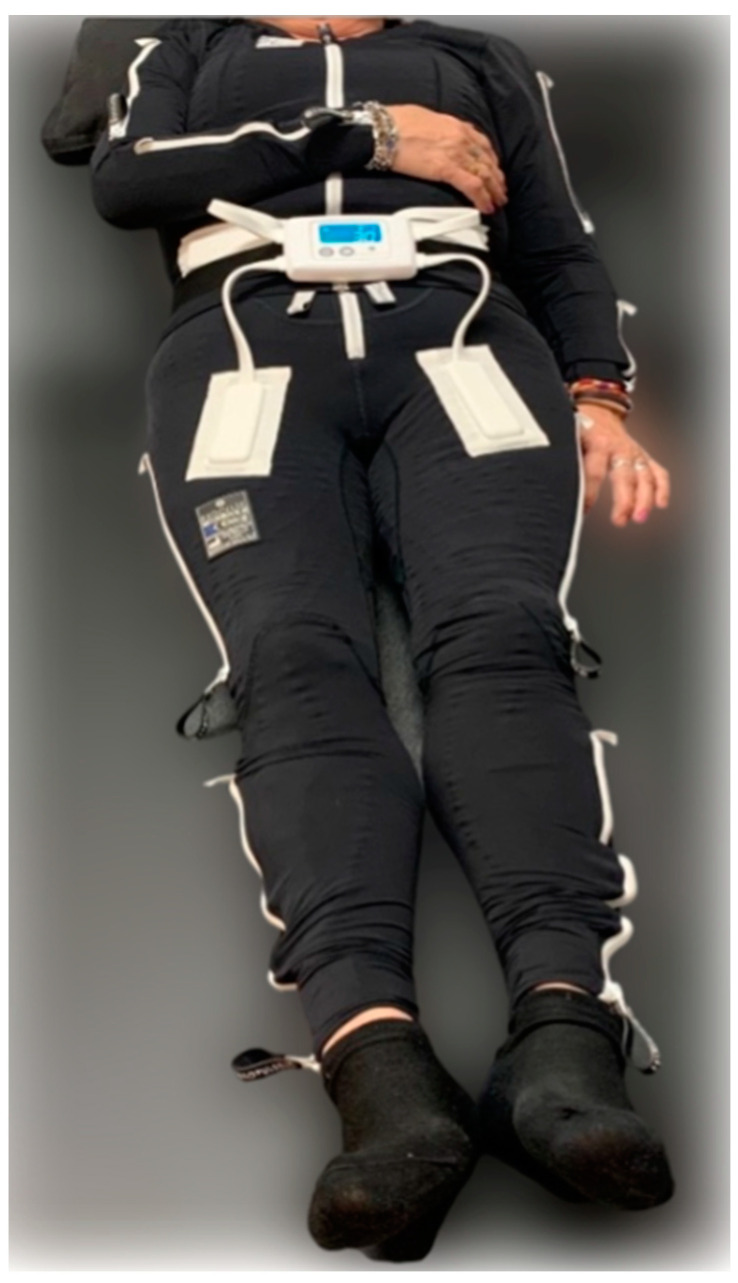
EXOPULSE Mollii Suit [88].

**Figure 2:**
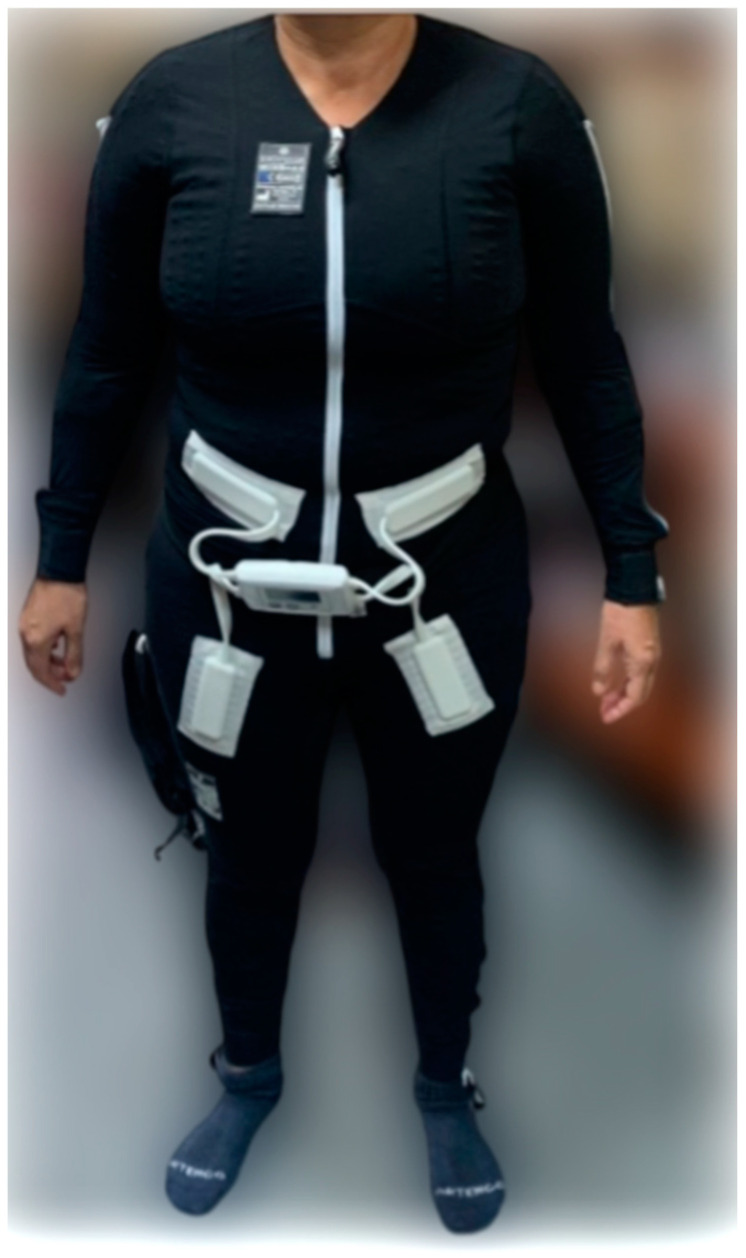
Mollii control unit [88].

To achieve the objectives of this study, participants were randomly allocated into four distinct groups: Suit, Suit + VR, Exercise, and Control. The procedural framework was consistent across all groups. Upon arrival at the study location, participants provided their written informed consent, followed by the completion of an initial baseline evaluation. Immediately subsequent to this baseline assessment, the designated intervention for each group was administered. At the conclusion of these interventions, a new set of measurements was taken to assess the immediate effects. The specific interventions administered to each group are detailed as follows:

- Suit: The same protocol used in our previous project [80,81,82,83,84,85,86] was carried out in this intervention. Therefore, participants in this group were subjected to a 60 min session with the EXOPULSE Mollii suit (Figure 1), with all 58 electrodes active at an intensity of 2 milliamperes (mA) and pulse width of 30 milliseconds (ms), as previously described in other studies using the same treatment [1,76,80,87]. In this line, after basal measurements, the participant suited up in the EXOPULSE Mollii suit with the help of a certified professional who ensured that all electrodes were correctly placed and were in contact with the patient’s skin. Once the suit was correctly placed, the control unit (Figure 2) was adhered to the suit, the patient was placed lying down and facing upwards on a massage table, the suit was turned on, and the session began. The patient was free to move throughout the session. Once the session was over, muscle oxygen levels and saliva recollection were carried out without taking off the suit. Afterwards, the patient changed clothes, and the rest of the post-session evaluation was carried out;
- Suit + VR: The same procedure as in the previous group was carried out; however, after placing the suit on the patient, they were given a VR visor (Oculus Go). After this, the patient lay down on the massage table facing upwards with the visor on. In this case, the patient could not move throughout the session. This group performed all sessions in an environment prepared for the VR protocol. Through the visor, the patient perceived the same surroundings as in the real-world environment where these sessions were performed, already registered in the visor. The patient saw herself lying down from her point of view in the form of a female avatar placed in the same position as the patient and wearing a suit similar to the EXOPULSE Mollii suit. During the session, the avatar gave commands as well as performed the movements commanded so the patient may mirror those movements. The patient performed two exercises, one with the upper extremities and the other one with the lower extremities, performing 2 sets of 10 repetitions with each extremity, with each set being followed by a brief period of rest where the patient was at liberty to take the visor off, as it could be a little overwhelming for some patients. The movements performed were as follows:
  ○ Upper extremities: starting from a position of adduction, internal rotation, hand closure and extension, the arm is brought to a position of flexion, abduction, and external rotation with opening of the hand;
  ○ Lower extremities: starting from a position of extension, adduction, and neutral rotation with one leg crossed over the other, the leg is brought to a position of flexion, abduction, and external rotation by sliding the heel through the opposite tibia;
- Exercise: The training group performed a 1 h training intervention consisting of a mobility warm up (Rate of Perceived Exertion (RPE) = 3) followed by a strength training session (RPE = 7) composed of 3 bodyweight exercises (adapted push-ups, sit-to-stand, and glute bridge) and 1 banded exercise (standing one-hand row). Participants performed 3 sets of 10 reps for each exercise with 1 min of rest between each set and exercise;
- Control: Participants in this group performed the same intervention as the Suit group; however, unbeknownst to the patients, the 58 electrodes of the suit were inactive.

### 2.4. Outcome Measures

Outcome measures were rigorously evaluated at two key points: prior to the initiation of treatment to establish baseline conditions and immediately following the intervention. This dual-phase evaluation methodology is consistent with approaches validated in previous studies, ensuring a reliable framework for assessing the effects of the interventions on the participants. The selection of variables and the methodologies employed for assessment were informed by established protocols delineated in prior research. This strategic approach not only facilitates the comparison of results across studies but also significantly contributes to the expanding body of scientific evidence regarding the efficacy of various treatment modalities for fibromyalgia [1]. Furthermore, incorporating standardized assessment techniques enhances this study’s validity and ensures that its findings are both credible and applicable to broader clinical practices.

#### 2.4.1. Thermography

Thermal imaging of the designated hot points was conducted using the advanced FLIR E8-XT thermography system [89]. This process entailed the acquisition of two precise measurements from both the dorsal (back) and palmar (front) sides of the hand. The FLIR E8-XT system, renowned for its high-resolution imaging and sensitivity, provided an automated and accurate assessment of thermal anomalies, facilitating the detection of subtle variations in skin temperature that are indicative of underlying physiological processes. This methodological choice underscores this study’s commitment to utilizing state-of-the-art technology to enhance the precision and reliability of its outcomes.

#### 2.4.2. Respiratory Variables

To measure the following variables, a spirometry test was conducted with a Vitalograph Asma1 spirometer [90]. The patient was asked to fully inhale (until the lungs were filled), close her lips around the mouthpiece, and exhale as quickly and forcefully as possible until the lungs were emptied, repeating this process 3 times [91]. The values of forced expiratory volume in 1 s (FEV1), 6 s (FEV6), and the ratio of both these values (FEV1/FEV6) [92] were registered. Further, chest perimeter difference between full air inspiration and full air expiration was also measured.

#### 2.4.3. Pain Severity

To assess pain severity, the Numeric Rating Scale (NRS) was used with a scale of 0–10, with 0 meaning “no pain” and 10 meaning “the worst pain imaginable” [93].

#### 2.4.4. Muscle Oxygen Variables

Muscle oxygen saturation (SmO_2_), total hemoglobin (THb), deoxygenated hemoglobin (HHb), and oxygenated hemoglobin (O_2_Hb) values were measured using a portable NIRS sensor (Moxy, Fortiori Design LLC, Hutchinson, MN, USA) connected with GoldenCheetah software (version 3.4, U.S.). This device, shown to be reliable at low and moderate intensity for the measuring of consumption of muscle oxygen (SmO_2_; ICC: r = 0.773–0.992) [94], was placed in the vast lateral quadriceps between the greater trochanter and the lateral femoral epicondyle. To reduce noise, a soft spline filter was applied using MATLAB^®^ software R2023b (The MathWorks, Inc., Natick, MA, USA). In the following sections, we used a second order 6 Hz cut-off Butterworth filter, applied two times to the time series.

#### 2.4.5. Cortical Arousal

Cortical arousal levels were quantified using the Critical Flicker Fusion Threshold (CFFT) method, executed within a controlled viewing chamber utilizing the Lafayette Instrument Flicker Fusion Control Unit Model 12021. This approach adheres to the procedural framework established in prior studies [95,96], ensuring methodological consistency and reliability. The CFFT technique involves determining the highest frequency at which an individual perceives a flickering light as being continuous, reflecting the brain’s processing speed and cortical activation. By following this established procedure, the study leverages a scientifically validated method to assess cortical arousal, contributing to the accuracy and comparability of the results within the broader context of neurophysiological research.

#### 2.4.6. Functional Test

Adapted from the exercise test battery by Carbonell-Baeza et al. (2022) for fibromyalgia patients [97], the functional tests used are as follows:

Chair stand test: the number of times within 30 s that an individual can rise to a full stand from a seated position with their back straight and feet flat on the floor, without pushing off with the arms;

Handgrip strength test: measured using a grip dynamometer (Kuptone, model EH101). Each patient performs the test twice with the dominant hand, with the arm fully extended, forming a 30° angle in relation to the trunk.

Ten meter up-and-go test: standing up from a chair and walking 10 m in the shortest time possible without running;

One-leg balance: the subject must keep balance on one leg with their eyes open as long as possible. This test is conducted on both legs.

#### 2.4.7. Pressure Pain Threshold

To accurately gauge participants’ general pain sensitivity, the Pressure Pain Threshold (PPT) was determined at two specific sites on the right side of the body, following protocols established in prior fibromyalgia research. These sites included the lateral epicondyle and the medial fat pad proximal to the knee joint line. During measurements, an algometer was applied perpendicularly to the skin at each site. Participants were instructed to verbally indicate the moment the pressure induced pain, thereby identifying the PPT. The algometer used for this study was calibrated to display pressure increases in increments of 0.01 kgf, ensuring precise measurement sensitivity and reliability in capturing the threshold at which pain was perceived by the participants. This increment size was chosen to balance measurement accuracy with participant comfort [98,99]. Additionally, the algometer was rigorously calibrated prior to the evaluations to guarantee the accuracy of the measurements, as corroborated by references [100,101]. This calibration process was critical to maintaining the integrity and consistency of the data collected throughout the study.

### 2.5. Statistical Analysis

In the statistical analysis section, we utilized SPSS (Statistical Package for Social Sciences, version 25, IBM, Armonk, NY, USA) for all analyses. Descriptive statistics were expressed as mean ± SD, and the normality of data distribution was verified using the Kolmogorov–Smirnov test. To evaluate the interventions’ effects, *t*-tests were applied to parametric variables, and the Wilcoxon test was used for non-parametric variables. For comparing effects between groups, a one-way ANOVA was conducted. The significance level was maintained at *p* ≤ 0.05 for all tests. Furthermore, to provide a comprehensive understanding of the intervention impacts, we calculated effect sizes using Cohen’s d formula. This metric helps quantify the difference between two means in terms of standard deviation, facilitating an interpretation of practical significance. According to Cohen’s benchmarks, effect sizes are categorized as small (d = 0.2), medium (d = 0.5), and large (d = 0.8). These thresholds were applied to interpret the magnitude of the treatment effects observed in our study, ensuring a clear understanding of their clinical relevance.

### 2.6. Ethical Aspects

The current study followed the ethical standards recognized by the Declaration of Helsinki [102], the EEC Good Clinical Practice recommendations (document 111/3976/88, July 1990), and current Spanish legislation regulating clinical and biomedical research in humans, personal data protection, and bioethics (Royal Decree 561/1993 on clinical trials and 14/2007, 3rd July, for Biomedical research). This study was explained to each participant before starting, and the participants voluntarily signed an informed consent form.

## 3. Results

Table 2 presents the sample characteristics. The sample was randomly divided into four different groups: Control (*n* = 20), Suit (*n* = 22), Suit + VR (*n* = 21), and Exercise (*n* = 21). The Control group presented a mean age of 55 years, while the other three groups presented a mean age between 51 and 52 years. Further, subjects presented a mean body mass index (BMI) of 27.6 for the Control group, 26 for the Suit group, 26.5 for the Suit + VR group, and 30.3 for the exercise group.

Table 3 presents the spirometry values and chest perimeter differences before and after the intervention. FEV 1, FEV 6, and FEV 1/FEV 6 had no significant changes prior to and after the intervention, except for FEV 1 values in the Suit + VR group, which experienced a decrease of 1.07 L after the intervention. Moreover, chest perimeter differences only had a significant increase of 0.65 cm and 0.54 after the Suit and Exercise interventions, respectively.

Muscle oxygen values are presented in Table 4. The subjects experienced a SmO_2_ increase of 1.52% in the Control group, 4.7% in the Suit group, 15.6% in the Suit + VR group, and 11.72% for the Exercise group. Further, a proportional change occurred in HHb and O_2_Hb values, as HHb decreased 1.97 and 1.43 g/dL while O_2_Hb values increased 1.77 and 1.34 g/dL, respectively, for the Suit + VR and Exercise groups.

Table 5 shows a generalized decrease in NRS values for all groups, with a nearly 1-point decrease in the Control group, 1.46-point decrease after the Suit intervention, 2.21-point decrease in the Suit + VR intervention, and 1.23-point decrease in the Exercise group. Additionally, PPT values had no significant changes, except for knee measurements for the Suit + VR and Exercise groups, with an increase of 0.61 and 0.42 kg, respectively.

Further, Table 6 shows cortical arousal and salivary patterns. Cortical arousal presented a significant increase of 1.4 Hz in the Control group and a 1.7 Hz increase in the Exercise group.

Functional test results are presented in Table 7. For the chair stand test, the subjects had significantly better results in the Suit (1.41 repetitions) and Exercise (1.58 repetitions) groups. Also, for the handgrip strength test, subjects in the intervention groups had a significant better performance, with a 0.53 kg decrease in the Control group and a 0.44 and 0.9 kg increase in both the Suit and Suit + VR groups. In addition, balance test values significantly increased in the Suit + VR group, by 11.79 s in the right leg, whilst decreasing by 12.15 s in the Suit group. And a significant increase occurred in the left leg in the Exercise group (13.28 s).

In Table 8, we can see the temperature values of the palm and back of the hand and the proximal and distal end of the index finger. All variables suffer a decrease in all intervention groups, except for the exercise group, where there is an increase in all variables after the training session.

In Table 9, results from the one-way ANOVA can be viewed, showing significant differences between the groups in post-intervention measurements for the following variables: PPT measured in both sites and all temperature and muscle oxygen variables. Further, between-group comparisons yielded the following significant differences: all temperature variables when comparing the Exercise group against the Suit and Suit + VR groups and all temperature variables except for dorsal temperature when comparing the Exercise group with Control; PPT values measured in the epicondyle between the Suit and the Control group and PPT knee values between the Control group and Suit + VR and Exercise; and all muscle oxygen variables between all groups. 

Table 10 shows the results of *t*-tests comparing pre- and post-intervention measurements of all variables in each group. Significant differences can be seen for the Control group in FEV 1/FEV 6, NRS, PPT performed in the epicondyle, cortical arousal, 10 m up-and-go test, and all temperature and muscle oxygen variables. Also, the Suit group had significant differences in the NRS, chair stand test, palm temperature, and all muscle oxygen variables. The Suit + VR group had significant differences in the NRS, PPT measured in the knee, handgrip strength test, 10 m up-and-go test, one-leg balance test with the right leg, SmO_2_, HHb, and O_2_Hb. Finally, the Exercise group had significant differences in FEV 1/FEV 6, chest perimeter difference, NRS, PPT measured in both the epicondyle and the knee, cortical arousal, chair stand test, 10 m up-and-go test, SmO_2_, HHb, and O_2_Hb.

Table 11 shows the results of normality assessment obtained after carrying out a Kolmogorov–Smirnov test on all variables. Samples are classified as having a parametric distribution if the *p* value is greater than 0.05 and classified as having a non-parametric distribution if the *p* value is inferior to 0.05.

## 4. Discussion

The objective of this study was to evaluate and compare the psychophysiological responses of fibromyalgia patients to different treatment modalities. Our hypothesis, positing significant differences in psychophysiological parameters between baseline (pre-intervention) and post-intervention assessments, was confirmed. Across all participant groups, significant variations in muscle oxygen saturation (SmO_2_), deoxygenated hemoglobin (HHb), and oxygenated hemoglobin (O_2_Hb) were observed both before and after the interventions, as well as between groups in the post-intervention phase. However, the magnitude of these effects, as measured by effect sizes, differed markedly across groups for all three measured variables between the pre- and post-intervention phases. Specifically, the Control and Suit groups did not reach the minimum effect size threshold of 0.4, in contrast to the Exercise and Suit + VR groups, which demonstrated substantial effect sizes ranging from 0.88–0.9 to 0.96–0.98 for all variables considered.

These outcomes align with prior findings on the application of the EXOPULSE Mollii suit in FM patients, which similarly reported significant increases in SmO_2_ and O_2_Hb levels alongside decreases in HHb [1]. Nonetheless, direct comparisons with other interventions were not feasible, given the innovative nature of muscle oxygenation measurements in the context of FM. Prior studies have suggested that FM patients typically exhibit lower SmO_2_ and O_2_Hb levels and higher HHb levels compared to healthy controls [103], a trend that was mirrored in our baseline data. It has been theorized that FM patients may experience mitochondrial dysfunction, leading to inadequate ATP production, as demonstrated by improvements in muscle oxygen values in earlier research [104] and corroborated by our findings. The utilization of the EXOPULSE Mollii suit appears to mitigate this condition, with further enhancements observed when the treatment is augmented with VR or complemented by a 1 h training session. This suggests that targeted interventions, particularly those incorporating advanced technological aids like the EXOPULSE Mollii suit and VR, may offer significant benefits in managing the physiological challenges associated with FM, potentially through mechanisms involving improved mitochondrial function and enhanced muscle oxygenation.

Pain perception in fibromyalgia patients has been linked to mitochondrial dysfunction, a condition that may be alleviated by observed improvements in muscle oxygenation, as indicated by significant changes in muscle oxygen saturation (SmO_2_), deoxygenated hemoglobin (HHb), and oxygenated hemoglobin (O_2_Hb) across all study groups [105]. This enhancement in muscle oxygenation coincides with a normalization of ATP production and a subsequent decrease in muscle oxygen demands, potentially leading to diminished pain perception among FM patients. Following the intervention, Numeric Rating Scale (NRS) scores significantly decreased in all groups, including the Control group, underscoring the subjective nature of pain perception and the significant role of patient-reported outcomes in assessing pain levels. The Control group exhibited a notably smaller effect size (0.5) in comparison to the other groups, which ranged from 0.7 to 0.8. However, despite these differences, there were no significant inter-group variations in NRS scores, highlighting the subjective and potentially placebo-influenced nature of this measurement.

In line with previous research [1,77], the Suit group experienced a significant reduction in subjective pain perception post intervention, a trend that was echoed in the training session group, though to a slightly lesser degree. These findings are consistent with earlier studies [106,107]. The Exercise group reported a decrease in the Pressure Pain Threshold (PPT) at both the knee and epicondyle locations, with an effect size of 0.6. Notably, the Control group also showed a significant reduction in epicondyle PPT, exhibiting a larger effect size than that observed in the Exercise group, suggesting a potential placebo effect influencing PPT measurements at this site. Both the Exercise and Suit + VR groups experienced significant reductions in knee PPT, with notable post-intervention differences when compared to the Control group, which aligns with prior research [1,108,109]. The Suit + VR group, in particular, demonstrated a more substantial decrease in NRS scores post intervention, indicated by a larger effect size, suggesting that combining the suit with VR exercises may have had an additive effect on reducing pain perception.

Upon conducting a retrospective analysis, no significant differences were observed in the majority of respiratory variables, with the exception of the FEV1/FEV6 ratio in the Control group and the percentile change in this ratio in both the Control and Exercise groups. However, subsequent inter-group comparisons post intervention failed to reveal any significant variations. The differences noted in the FEV1/FEV6 ratio, yielding effect sizes of approximately 0.6 in the Control group and 0.4 in percentile change within the Exercise group, suggest only minimal respiratory alterations attributable to the interventions in fibromyalgia patients. Although these effect sizes are statistically significant, they do not imply major physiological changes as a result of the applied treatment modalities. Interestingly, a post-intervention increase in chest perimeter difference was observed in the Exercise group, with an effect size of 0.4. This modification may be attributed to improved costal mobility and increased activity in both expiratory and inspiratory muscles, reflecting a physiological adaptation to the elevated oxygen demands during exercise. This indicates that while the interventions may not significantly impact fundamental respiratory parameters, exercise can provoke specific thoracic adjustments that enhance respiratory functionality in FM patients.

In this study, responses to functional tests post intervention showed more pronounced variability among groups compared to other evaluated variables. Notably, the “10 m up-and-go” test revealed significant reductions in completion time for the Control, Suit + VR, and Exercise groups, with effect sizes of 0.7, 0.8, and 0.5, respectively. The relatively large effect size in the Control group, in comparison to the Exercise group, might imply a placebo effect. However, the considerable effect size observed in the Suit + VR group deserves particular attention. Moreover, the Suit + VR intervention led to notable improvements in handgrip strength and right-leg balance, with effect sizes of 0.4 and 0.7, respectively. Similarly, both the Suit and Exercise groups experienced significant enhancements in the chair stand test, with effect sizes of 0.5 and 0.7, respectively. Yet, these improvements in functional tests did not show significant differences between groups.

It is essential to compare these findings with prior research [1], which reported improvements in all functional tests following a session utilizing the EXOPULSE Mollii suit for a single FM patient. Our broader study only demonstrated enhancements in specific functional variables, suggesting potential variability in response to the intervention. This discrepancy highlights the necessity for additional research to fully comprehend these outcomes. The observed improvements in the Exercise group could be ascribed to increased muscle activation, leading to improved strength output and better performance in the chair stand test and a reduced completion time in the “10 m up and go” test. This suggests that more prolonged or intense exposure to exercise stimuli may be required to elicit more substantial functional adaptations in FM patients, potentially enhancing their quality of life. These insights set the stage for future investigations into the long-term functional effects of various interventions on FM patients [106,107].

Aligned with prior research, it has been established that patients with fibromyalgia typically exhibit higher levels of sympathetic nervous system (SNS) activation under baseline conditions compared to healthy controls [110,111]. In our investigation, a decrease in the temperature of the hand and index finger was noted across all groups, with the exception of the Exercise group. This exception is likely due to the enhanced blood flow necessitated by muscular activity during exercise, which naturally leads to an increase in body temperature. The temperature reductions observed in the Control, Suit, and Suit + VR groups were statistically significant for both the Control and Suit groups in terms of hand temperature. These findings are in concordance with previous studies that noted temperature decreases post treatment with the EXOPULSE Molli suit [1], indicative of reduced peripheral blood flow and suggesting a shift towards increased parasympathetic tone and a decrease in SNS activity [1,112]. This shift in autonomic balance is also thought to be associated with the decreases in pain perception reported among these groups.

Moreover, significant increases in cortical arousal values were observed in both the Control and Exercise groups, with effect sizes of 0.7 and 0.99, respectively. These increases suggest a pronounced enhancement in parasympathetic activity, possibly serving as a compensatory mechanism to counteract the intense SNS activation observed during the exercise intervention. This finding prompts further investigation, especially regarding the duration and consistency of this elevated parasympathetic response after repeated treatment sessions. Additionally, the integration of heart rate variability (HRV) measurements into future research could offer more comprehensive insights into the autonomic nervous system’s responses to different interventions in fibromyalgia patients, enriching our understanding of the condition’s complex pathophysiology and potential therapeutic avenues.

## 5. Conclusions

In conclusion, our research presents compelling evidence that the EXOPULSE Mollii suit, both alone and in combination with virtual reality (VR), as well as a dedicated 1 h training session serve as effective treatment modalities for fibromyalgia (FM) patients, each yielding acute beneficial impacts. Notably, the augmented effects observed when the EXOPULSE Mollii suit is paired with VR, or when a comprehensive 1 h training session is implemented, highlight their superior efficacy over the use of the suit in isolation. This insight holds particular significance for FM patients grappling with severe pain and fatigue, showcasing the standalone suit as a viable treatment option while also suggesting enhanced benefits through its combination with other interventions.

The potential for a synergistic effect through the combination of these treatment approaches merits further investigation. Future research could explore various integration strategies, such as alternating between modalities or combining them within a cohesive treatment program. For example, assessing the impact of consecutive sessions involving the EXOPULSE Mollii suit followed by physical training, or the inclusion of high-intensity exercises during suit use, could yield critical insights into refining treatment protocols for FM patients.

Crucially, the interventions evaluated in this study demonstrated significant improvements in essential aspects such as muscle oxygenation, subjective pain perception, and activation of the parasympathetic nervous system. These outcomes highlight the comprehensive benefits of these treatments in addressing the complex symptom profile of FM, offering a multidimensional approach to management.

### Practical Applications and Future Lines of Research

To advance the management of fibromyalgia, future investigations should prioritize longitudinal studies that assess the enduring impacts of neuromodulation, virtual reality, and exercise interventions on patient outcomes. Conducting randomized controlled trials to compare these innovative treatments to conventional care is essential, with a focus on evaluating their effectiveness in providing symptom relief, enhancing quality of life, and improving functional abilities. Furthermore, delving into the biological mechanisms that underlie the response of fibromyalgia to these interventions may reveal new therapeutic targets, offering a pathway to more effective treatments.

In clinical practice, the insights gained from this research study offer valuable guidance for healthcare providers. By integrating findings from this study, clinicians can develop personalized treatment strategies that incorporate a multimodal approach, aligning with the unique needs and preferences of each patient. Such tailored treatment plans promise to improve patient care by offering targeted interventions that address the multifaceted nature of fibromyalgia, potentially leading to better health outcomes and enhanced quality of life for patients.

This approach underscores the importance of embracing a comprehensive perspective in fibromyalgia management, highlighting the need for ongoing research to refine and expand treatment options. Future studies should also explore the potential synergies between different therapeutic modalities, assessing their combined effects on fibromyalgia symptoms and patient well-being. By continuing to investigate and innovate, we can move closer to optimizing treatment strategies for fibromyalgia, ultimately contributing to more effective and personalized patient care.

## Figures and Tables

**Table 1:** Inclusion and exclusion criteria.

Inclusion Criteria	Exclusion Criteria
Diagnosed with fibromyalgia by a rheumatologist according to the ACR criteria with a minimum of 3 months since diagnosis. Female. Between 18 and 67 years of age. Able to walk independently without assistive devices. Not participating in other clinical trials during the study period. Has not received neuromodulation therapy or participated in structured exercise programs in the last 6 months.	Failure to complete written consent. Presence of other neurological disorders or conditions that significantly affect pain perception. Recent surgery or injury to the musculoskeletal system within the past 6 months. Severe cardiovascular or respiratory conditions that contraindicate exercise. Current use of opioids or changes in medication regimen that could affect pain perception within the last month.

**Table 2:** Descriptive characteristics of patients.

Variable	Control	Suit	Suit + VR	Exercise
Number, *n*	20	22	21	26
Age, y	55.1 ± 8.34	51.8 ± 8.37	51 ± 10.75	51.5 ± 10.91
Weight, kg	71 ± 10.22	66.5 ± 9.18	67.8 ± 12.27	79.5 ± 13.76
Height, cm	160.8 ± 6.28	160.1 ± 5.39	160 ± 6.67	161.9 ± 6.37
Body mass index, kg/m^2^	27.6 ± 4.49	26 ± 3.41	26.5 ± 4.62	30.3 ± 4.52

Data are expressed as means ± SD for quantitative variables. VR: virtual reality.

**Table 3:** Respiratory values of the sample before and after the intervention for each group.

Variables	Control		Suit		Suit + VR		Exercise	
	Pre	Post	Pre	Post	Pre	Post	Pre	Post
FEV 1, L	2.21 ± 0.38	2.24 ± 0.38	2.24 ± 0.50	2.17 ± 0.50	3.91 ± 8.07	2.24 ± 0.46	2.21 ± 0.58	2.25 ± 0.59
FEV 1, %	93.80 ± 17.77	94.60 ± 14.53	90.80 ± 15.70	87.48 ± 18.42	88.00 ± 17.65	91.00 ± 16.29	89.44 ± 18.75	90.84 ± 19.45
FEV 6, L	2.64 ± 0.31	2.55 ± 0.41	2.70 ± 0.56	2.64 ± 0.64	3.87 ± 6.26	2.59 ± 0.52	2.59 ± 0.68	2.62 ± 0.65
FEV 6, %	94.75 ± 11.71	91.05 ± 12.38	93.00 ± 13.78	89.62 ± 17.06	86.79 ± 17.62	89.58 ± 14.84	88.92 ± 18.54	90.08 ± 17.99
FEV 1/FEV 6, *n*	0.84 ± 0.10	0.88 ± 0.05	0.83 ± 0.09	0.83 ± 0.08	0.88 ± 0.11	0.86 ± 0.07	0.86 ± 0.06	0.86 ± 0.08
FEV 1/FEV 6, %	106.30 ± 11.85	111.55 ± 5.83	105.55 ± 12.45	104.71 ± 10.53	108.90 ± 7.89	108.79 ± 9.02	108.24 ± 8.59	108.88 ± 11.24
Chest perimeter difference, cm	7.40 ± 2.55	7.48 ± 1.97	6.65 ± 2.16	7.30 ± 1.72	6.49 ± 2.79	6.79 ± 2.75	6.28 ± 1.94	6.82 ± 2.21

Data are expressed as means ± SD for quantitative variables. FEV 1: forced expiratory volume in 1 s. FEV 6: forced expiratory volume in 6 s.

**Table 4:** Muscle oxygen values of the sample before and after the intervention for each group.

Variables	Control		Suit		Suit + VR		Exercise	
	Pre	Post	Pre	Post	Pre	Post	Pre	Post
SmO_2_, %	48.63 ± 11.53	50.15 ± 12.25	53.2 ± 16.12	57.94 ± 15.50	45.76 ± 15.05	61.34 ± 12.36	43.91 ± 14.86	55.63 ± 17.62
THb, g/dL	11.85 ± 0.36	11.96 ± 0.32	11.76 ± 0.34	11.85 ± 0.44	12.01 ± 0.39	11.81 ± 0.35	11.92 ± 0.40	11.82 ± 0.45
HHb, g/dL	6.11 ± 1.47	5.98 ± 1.55	5.52 ± 1.95	5.01 ± 1.93	6.54 ± 1.87	4.57 ± 1.51	6.71 ± 1.87	5.28 ± 2.16
O_2_Hb, g/dL	5.74 ± 1.27	5.97 ± 1.37	6.23 ± 1.82	6.84 ± 1.74	5.47 ± 1.70	7.24 ± 1.45	5.21 ± 1.72	6.55 ± 1.99

Data are expressed as means ± SD for quantitative variables. SmO_2_: muscle oxygen saturation. THb: total hemoglobin. HHb: deoxygenated hemoglobin. O_2_Hb: oxygenated hemoglobin.

**Table 5:** NRS and PPT values of the sample before and after the intervention for each group.

Variables	Control		Suit		Suit + VR		Exercise	
	Pre	Post	Pre	Post	Pre	Post	Pre	Post
NRS, 0–10	7.1 ± 1.714	6.15 ± 1.981	5.91 ± 1.63	4.45 ± 2.425	6.76 ± 1.814	4.55 ± 2.743	6.58 ± 1.88	5.35 ± 2.56
PPT epicondyle, kg	1.24 ± 0.527	1.55 ± 0.561	2.04 ± 1.183	2.05 ± 0.665	1.78 ± 0.683	1.78 ± 0.504	1.9 ± 0.897	2.17 ± 1.09
PPT knee, kg	1.86 ± 1.851	1.8 ± 0.833	2.1 ± 0.861	2.25 ± 0.811	2.02 ± 0.973	2.63 ± 1.25	2.09 ± 0.951	2.51 ± 1.232

Data are expressed as means ± SD for quantitative variables. NRS: Numeric Rating Scale. PPT: Pressure Pain Threshold.

**Table 6:** Cortical arousal and saliva values of the sample before and after the intervention for each group.

Variables	Control		Suit		Suit + VR		Exercise	
	Pre	Post	Pre	Post	Pre	Post	Pre	Post
Cortical Arousal, Hz	33.8 ± 2.4	35.2 ± 3.54	33.7 ± 3.65	33.5 ± 2.84	33.2 ± 2.28	34 ± 2.9	32.6 ± 2.47	34.3 ± 2.82

Data are expressed as means ± SD for quantitative variables.

**Table 7:** Functional test results of the sample before and after the intervention for each group.

Variables	Control		Suit		Suit + VR		Exercise	
	Pre	Post	Pre	Post	Pre	Post	Pre	Post
Chair stand test, *n*	11.35 ± 5.12	11.15 ± 3.63	15.41 ± 6.98	16.82 ± 7.66	12.71 ± 3.36	13.24 ± 4.85	13.46 ± 7.38	15.04 ± 8.07
Handgrip strength test, kg	22.53 ± 4.54	22 ± 4.93	22.57 ± 5.15	23.01 ± 5.77	23.14 ± 5.05	24.04 ± 5.39	23.12 ± 4.50	23.32 ± 4.10
10 m up-and-go test, s	7.46 ± 2.73	7.02 ± 2.63	6.33 ± 1.57	6.13 ± 1.47	6.17 ± 0.93	5.75 ± 0.99	6.37 ± 1.29	6.08 ± 1.13
One-leg balance (right), s	22.93 ± 17.25	26.75 ± 23.32	54.04 ± 48.68	41.89 ± 26.29	28.51 ± 28.56	40.3 ± 35.09	43.79 ± 42.62	49.97 ± 49.36
One-leg balance (left), s	19.59 ± 17.86	25.11 ± 21.21	43.06 ± 32.58	45.57 ± 37.37	33.26 ± 28.63	35.62 ± 33.79	45.51 ± 39.24	58.79 ± 69.17

Data are expressed as means ± SD for quantitative variables.

**Table 8:** Hand and index finger temperature values of the sample before and after the intervention for each group.

Variables	Control		Suit		Suit + VR		Exercise	
	Pre	Post	Pre	Post	Pre	Post	Pre	Post
Palm Tª, °C	32.8 ± 1.97	31.8 ± 2.24	32.6 ± 3.54	31.2 ± 2.69	32 ± 2.91	31.2 ± 2.97	33 ± 2.29	34.2 ± 2.13
Dorsal Tª, °C	32 ± 2.00	31.2 ± 2.21	31.8 ± 3.45	30.7 ± 2.87	31.1 ± 2.58	31.1 ± 2.48	32.3 ± 2.50	33 ± 2.27
Proximal index finger Tª, °C	31.6 ± 3.13	30.3 ± 3.09	31 ± 4.79	29.5 ± 3.42	30.5 ± 3.76	29.6 ± 3.73	30.6 ± 7.23	33.7 ± 2.65
Distal index finger Tª, °C	30.7 ± 3.76	29.3 ± 3.16	29.3 ± 5.21	28.3 ± 3.47	29.1 ± 4.43	27.8 ± 3.89	29.8 ± 7.11	32.4 ± 3.81

Data are expressed as means ± SD for quantitative variables. Tª, temperature.

**Table 9:** Comparative statistics of post-intervention results between groups.

Variables	*p*	ε^2^	Suit vs. Suit + VR	Suit vs. Exercise	Suit vs. Control	Suit + VR vs. Exercise	Suit + VR vs. Control	Exercise vs. Control
FEV 1, L	0.909	0.006	0.922	0.972	0.964	1	0.957	0.991
FEV 1, %	0.629	0.021	0.973	0.937	0.593	0.998	0.879	0.803
FEV 6, L	0.967	0.003	1	0.998	0.98	0.993	0.988	0.966
FEV 6, %	0.968	0.003	0.998	1	1	0.989	0.97	0.937
FEV 1/FEV 6, *n*	0.054	0.088	0.241	0.15	0.052	1	0.976	0.885
FEV 1/FEV 6, %	0.056	0.090	0.318	0.168	0.051	1	0.84	0.864
Chest perimeter difference, cm	0.4	0.034	0.574	0.712	1	0.986	0.547	0.687
NRS, 0–10	0.09	0.075	0.999	0.667	0.069	0.773	0.227	0.604
PPT epicondyle, kg	0.037 *	0.101	0.491	0.987	0.044 *	0.759	0.491	0.102
PPT knee, kg	0.008 *	0.140	0.884	0.99	0.151	0.903	0.004 *	0.048 *
Cortical arousal, Hz	0.471	0.029	0.941	0.723	0.462	0.959	0.7	0.979
Chair stand test, *n*	0.094	0.073	0.38	0.65	0.072	0.969	0.737	0.505
Handgrip strength test, kg	0.552	0.024	0.83	0.996	0.949	0.853	0.469	0.894
10 m up-and-go test, s	0.255	0.046	0.914	0.964	0.533	0.587	0.226	0.861
One-leg balance (right), s	0.201	0.053	0.914	0.978	0.144	0.965	0.561	0.406
One-leg balance (left), s	0.069	0.082	0.494	1	0.197	0.367	0.838	0.119
Palm Tª, °C	<0.001 *	0.211	0.993	0.003 *	0.827	0.005 *	0.716	0.007 *
Dorsal Tª, °C	0.008 *	0.135	0.98	0.032 *	0.915	0.034 *	0.976	0.051
Proximal index finger Tª, °C	<0.001 *	0.251	0.999	<.001 *	0.869	0.003 *	0.791	0.003 *
Distal index finger Tª, °C	<0.001 *	0.219	0.957	0.003 *	0.788	0.002 *	0.535	0.011 *
SmO_2_, %	<0.001 *	0.086	<0.001 *	<0.001 *	<0.001 *	<0.001 *	<0.001 *	<0.001 *
THb, g/dL	<0.001 *	0.034	0.005 *	<0.001 *	<0.001 *	0.214	<0.001 *	<0.001 *
HHb, g/dL	<0.001 *	0.082	<0.001 *	<0.001 *	<0.001 *	<0.001 *	<0.001 *	<0.001 *
O_2_Hb, g/dL	<0.001 *	0.086	<0.001 *	<0.001 *	<0.001 *	<0.001 *	<0.001 *	<0.001 *

* *p* < 0.05. FEV 1: forced expiratory volume in 1 s. FEV 6: forced expiratory volume in 6 s. NRS: Numeric Rating Scale. PPT: Pressure Pain Threshold. SmO_2_: muscle oxygen saturation. THb: total hemoglobin. HHb: deoxygenated hemoglobin. O_2_Hb: oxygenated hemoglobin. Tª, temperature.

**Table 10:** Comparative statistics of pre- and post-intervention measurements for each group.

Variables	Control		Suit		Suit + VR		Exercise	
	*p*	Effect Size	*p*	Effect Size	*p*	Effect Size	*p*	Effect Size
FEV 1, L	0.292	0.124	0.865	0.247	0.537	0.019	0.2	0.171
FEV 1, %	0.357	0.083	0.812	0.203	0.211	0.189	0.221	0.156
FEV 6, L	0.974	0.462	0.89	0.276	0.507	0.000	0.231	0.149
FEV 6, %	0.979	0.486	0.911	0.313	0.173	0.222	0.193	0.177
FEV 1/FEV 6, *n*	0.005 *	0.648	0.671	0.108	0.514	0.004	0.051	0.379
FEV 1/FEV 6, %	0.005 *	0.674	0.661	0.105	0.37	0.098	0.043 *	0.419
Chest perimeter difference, cm	0.462	0.033	0.086	0.302	0.162	0.220	0.019 *	0.440
NRS, 0–10	0.033 *	0.582	0.002 *	0.778	0.002 *	0.817	0.002 *	0.723
PPT epicondyle, kg	<0.001 *	0.868	0.293	0.157	0.088	0.368	0.013 *	0.602
PPT knee, kg	0.583	0.047	0.261	0.183	0.01 *	0.591	0.007 *	0.615
Cortical arousal, Hz	0.002 *	0.724	0.636	0.082	0.037	0.411	<0.001 *	0.998
Chair stand test, *n*	0.273	0.170	0.012 *	0.515	0.24	0.206	0.002 *	0.727
Handgrip strength test, kg	0.914	0.343	0.259	0.140	0.041 *	0.398	0.343	0.080
10 m up-and-go test, s	0.002 *	0.724	0.052	0.399	<0.001 *	0.853	0.006 *	0.536
One-leg balance (right), s	0.565	0.038	0.788	0.195	<0.001 *	0.758	0.087	0.320
One-leg balance (left), s	0.147	0.276	0.431	0.048	0.329	0.117	0.087	0.320
Palm Tª, °C	0.007 *	0.600	0.04 *	0.431	0.068	0.395	0.997	0.619
Dorsal Tª, °C	0.018 *	0.505	0.102	0.337	0.504	0.002	0.994	0.583
Proximal index finger Tª, °C	0.013 *	0.537	0.079	0.348	0.125	0.258	0.998	0.647
Distal index finger Tª, °C	0.006 *	0.629	0.192	0.221	0.06	0.354	0.988	0.501
SmO_2_, %	<0.001 *	0.105	<0.001 *	0.386	<0.001 *	0.974	<0.001 *	0.892
THb, g/dL	<0.001 *	0.351	<0.001 *	0.337	1	0.608	1	0.387
HHb, g/dL	0.009 *	0.046	<0.001 *	0.346	<0.001 *	0.979	<0.001 *	0.880
O_2_Hb, g/dL	<0.001 *	0.165	<0.001 *	0.430	<0.001 *	0.968	<0.001 *	0.889

* *p* < 0.05. FEV 1: forced expiratory volume in 1 s. FEV 6: forced expiratory volume in 6 s. NRS: Numeric Rating Scale. PPT: Pressure Pain Threshold. SmO_2_: muscle oxygen saturation. THb: total hemoglobin. HHb: deoxygenated hemoglobin. O_2_Hb: oxygenated hemoglobin. Tª, temperature.

**Table 11:** Normality assessment of the sample.

Variables	Control		Suit		Suit + VR		Exercise	
	Pre	Post	Pre	Post	Pre	Post	Pre	Post
FEV 1, L	0.662	0.215	0.934	0.935	<0.001	0.384	0.594	0.644
FEV 1, %	0.672	0.659	0.996	0.23	0.086	0.97	0.437	0.272
FEV 6, L	0.097	0.798	0.513	0.827	<0.001	0.847	0.893	0.644
FEV 6, %	0.17	0.876	0.452	0.161	0.291	0.587	0.692	0.277
FEV 1/FEV 6, *n*	0.022	0.524	<0.001	0.02	<0.001	0.001	0.008	<0.001
FEV 1/FEV 6, %	0.043	0.606	<0.001	0.043	0.011	0.018	0.103	0.002
Chest perimeter difference, cm	0.867	0.024	0.136	0.14	0.128	0.56	0.451	0.525
NRS, 0–10	0.008	0.006	0.042	0.082	0.004	0.433	<0.001	0.716
PPT epicondyle, kg	0.006	0.725	<0.001	0.112	0.012	0.2	<0.001	<0.001
PPT knee, kg	<0.001	<0.001	0.107	0.302	0.01	<0.001	0.002	0.004
Cortical arousal, Hz	0.023	<0.001	0.06	0.18	0.931	0.16	0.154	0.717
Chair stand test, *n*	0.003	0.496	0.489	0.198	0.373	0.007	<0.001	<0.001
Handgrip strength test, kg	0.095	0.019	0.572	0.161	0.194	0.053	0.999	0.389
10 m up-and-go test, s	<0.001	<0.001	0.002	0.014	0.01	0.11	0.217	0.744
One-leg balance (right), s	0.008	0.003	0.007	0.091	<0.001	0.005	<0.001	<0.001
One-leg balance (left), s	0.014	0.017	0.023	0.004	0.007	0.001	<0.001	<0.001
Palm Tª, °C	0.141	0.926	<0.001	0.166	0.04	0.255	0.001	0.021
Dorsal Tª, °C	0.634	0.869	0.005	0.483	0.335	0.364	0.008	0.236
Proximal index finger Tª, °C	0.055	0.987	0.008	0.27	0.293	0.346	<0.001	0.004
Distal index finger Tª, °C	0.029	0.399	0.01	0.232	0.071	0.641	<0.001	0.001
SmO_2_, %	<0.001	<0.001	<0.001	<0.001	<0.001	<0.001	<0.001	<0.001
THb, g/dL	<0.001	<0.001	<0.001	<0.001	<0.001	<0.001	<0.001	<0.001
HHb, g/dL	<0.001	<0.001	<0.001	<0.001	<0.001	<0.001	<0.001	<0.001
O_2_Hb, g/dL	<0.001	<0.001	<0.001	<0.001	<0.001	<0.001	<0.001	<0.001

For normality assessment values, >0.05 indicates a parametric distribution of the sample and <0.05 indicates a non-parametric distribution of the sample. FEV 1: forced expiratory volume in 1 s. FEV 6: forced expiratory volume in 6 s. NRS: Numeric Rating Scale. PPT: Pressure Pain Threshold. SmO_2_: muscle oxygen saturation. THb: total hemoglobin. HHb: deoxygenated hemoglobin. O_2_Hb: oxygenated hemoglobin. Tª, temperature.

## Data Availability

Data are contained within the article.
